# Editorial: Model organisms in respiratory pharmacology 2023

**DOI:** 10.3389/fphar.2024.1540222

**Published:** 2025-01-13

**Authors:** Alexandra McCarron, Ranu Surolia, Livia Ruffini

**Affiliations:** ^1^ Adelaide Medical School, Faculty of Health and Medical Sciences, The University of Adelaide, Adelaide, SA, Australia; ^2^ Department of Respiratory and Sleep Medicine, Robinson Research Institute, University of Adelaide, Adelaide, SA, Australia; ^3^ Women’s and Children’s Hospital, North Adelaide, SA, Australia; ^4^ Division of Pulmonary, Allergy and Critical Care, Department of Medicine, University of Alabama at Birmingham, Birmingham, AL, United States; ^5^ Nuclear Medicine Division, University Hospital of Parma, Parma, Italy

**Keywords:** lung, respiratory, pharmacology, cell culture, animal model, computational modeling

Respiratory diseases remain the third leading cause of mortality worldwide. Chronic lung diseases affect approximately half a billion people globally with four million deaths each year (Momtazmanesh et al., 2023). Respiratory conditions inflict significant impact on individual quality of life, with many experiencing disabling symptoms including breathlessness and fatigue. Those affected often experience social isolation, have limited capacity for physical activity and are more likely to experience mental health conditions such as anxiety (Booth and Johnson, 2019).

Treatment of lung diseases places a significant burden on healthcare systems, particularly because many remain incurable, necessitating high levels of care for symptom management. Research models that accurately recapitulate human lungs and various disease states are essential for advancing the field. Improving our fundamental understanding of the pathogenesis of lung diseases is essential for identifying new therapeutic targets. Development of effective treatments, management strategies, preventive approaches, as well as diagnostic and disease monitoring tools are all necessary to improve the clinical care of lung conditions.

Our recent global experiences with the COVID-19 pandemic reinforces the ongoing need for accurate respiratory models. During the early stages of the pandemic the scientific community rapidly mobilised to develop cell culture and animal models to elucidate mechanisms of COVID-19 pathogenesis (Leist et al., 2020). This was essential for enabling testing of candidate vaccines and anti-viral drugs for rapid translation to human clinical trials.

To advance research, accurate recapitulation of the human respiratory system using biological models such as animals and cell cultures is essential. Modelling a variety of respiratory diseases is also crucial for elucidating causative mechanisms, identifying novel biomarkers and for testing candidate treatments. This is a continually advancing area of research, particularly because many existing model platforms have inherent limitations. A major advancement in the field has been the widespread adoption of *in vitro* systems including air-liquid interface cultures and airway organoids, which more closely replicate the cellular architecture and physiology of the airways when compared to standard monolayer cultures (Prytherch et al., 2011; Sachs et al., 2019). Equally important is the emerging role of computational biology. Computational models integrate structural, functional, and dynamic data of the respiratory system to simulate various physiological and pathological conditions, allowing for predictions of disease progression, response to therapies, and personalised treatment outcomes.

This Research Topic gathers five original articles that provide insights into the generation and use of respiratory models. This includes using animal models to assess novel treatment strategies (Song et al., Ge et al.), characterising animal model utility in the response to clinical pharmaceuticals (Reyne et al.), identifying novel biomarkers for use in disease diagnosis and monitoring (Mao et al.), and using computational approaches to model the lungs (Liu et al.).

Acute lung injury (ALI) is an inflammatory disease that causes disruption to the lung vascular endothelium and alveolar epithelium, leading to impairment in oxygen and carbon dioxide transfer. ALI can be caused by sepsis, pneumonia, trauma, aspiration, blood transfusions, pancreatitis and inhalation of smoke or toxic gas. Acute respiratory distress syndrome (ARDS) is the more severe form of ALI, and is associated with high levels of mortality (∼40%). Supportive care including supplementary oxygen and mechanical ventilation remain the primary treatment options. Despite several clinical trials, pharmaceutical therapies have not yet been found to have an impact on reducing mortality (Johnson and Matthay, 2010).

In this Research Topic, two pharmaceutical approaches were assessed for the treatment of ALI/ARDS. Ge et al. targeted extracellular histones as a therapeutic option for ARDS (Ge et al.). Neutrophils play a major role in eliminating pathogens and do so via a range of mechanisms. One approach is the formation of neutrophil extracellular traps, which consist of filamentous DNA and protein particles, of which histones are a large contributor. Excessive release of histones have been found to augment lung tissue injury and is linked with poorer prognosis in ARDS. Therapeutic strategies that neutralise and degrade histones may therefore prove effective for treatment of ARDS (Karki et al., 2020). Ge et al. investigated the use of polyanion molecule STC3141, which has previously been shown to neutralise histones. STC3141 was assessed in a lipopolysaccharide-induced rat model of ALI, where it was found to significantly reduce white blood cell and neutrophil counts, improve oxygenation and reduce lung inflammation (Sachs et al., 2019).

Song et al. examined a different approach for the treatment of ALI/ARDS, which involves blocking calcium to inhibit macrophage activation (Song et al.). Macrophages are important in regulating inflammatory responses. They secrete pro-inflammatory cytokines that recruit neutrophils, leading to prolonged and excessive responses that contribute to lung tissue destruction. Therefore, inhibition of macrophages may be a useful target for ALI treatment. Blocking calcium has been previously shown to reduce inflammation. Lomerizine is a drug that blocks calcium channels and is used clinically to treat migraine (Ikeda et al., 2018). In the study conducted by Song et al. lomerizine was found to inhibit inflammation and lung injury in lipopolysaccharide-induced ALI mice. *In vitro* studies examined the mechanism of action of lomerizine and found that blocking calcium influx in macrophages significantly reduces the expression of pro-inflammatory cytokines, thereby reducing neutrophil recruitment. Both papers demonstrate potential pharmaceutical agents for further exploration in the treatment of ALI/ARDS.

Shifting direction to inherited lung diseases, cystic fibrosis (CF) is a genetic condition that results in respiratory disease characterised by frequent infections, inflammation, muco-obstruction of the airways, and progressive loss of lung function. The disease arises from mutations in the cystic fibrosis transmembrane conductance regulator (*CFTR*) gene, which encodes for an epithelial cell chloride channel. When the *CFTR* gene is mutated, there is disruption to chloride secretion, sodium absorption and water transport in the airways, leading to accumulation of thick mucus (Turcios, 2020). Modulator drugs have become the gold-standard therapy for those with amenable *CFTR* variants. These medications work directly on the CFTR protein to restore ion channel function (Lopes-Pacheco, 2019). Modulator therapy improves lung health outcomes by preserving lung function and reducing the frequency of exacerbations (Middleton et al., 2019; Heijerman et al., 2019). Notably, there is a proportion of individuals with CF (∼10%) who are not eligible for modulator therapy as they carry *CFTR* variants that cannot be rescued with this pharmaceutical approach (Lopes-Pacheco, 2019).

Elexacaftor-tezacaftor-ivacaftor (ETI) is a CFTR modulator combination used to treat the Phe508del mutation, which is the most common disease-causing gene variant, affecting 90% of the CF population (Lopes-Pacheco, 2019). Reyne et al. investigated whether CF rats with the Phe508del mutation were responsive to ETI (Reyne et al.; McCarron et al., 2020). Assessment of CFTR ion channel function in the nasal epithelium via nasal potential difference (NPD) showed there was significant restoration in CFTR activity following oral treatment with ETI. Examination of respiratory mechanics (via flexiVent) and lung ventilation (via X-ray velocimetry) revealed no changes following ETI therapy. This work demonstrates the utility of an ETI-responsive CF Phe508del rat model for research applications, including assessing the impact of these drugs on disease trajectory, as well as side effects associated with their long-term use (Reyne et al.).

Exosomes are extracellular vesicles secreted by many cell types and play a role in cellular communication, homeostasis, and more recently have been found to contribute to inflammatory processes in lung diseases such as CF, chronic obstructive pulmonary disease (COPD) and asthma. Mao et al. found that exosomes carry disease-relevant protein signatures (Mao et al.). The *Scnn1b* transgenic mouse model of CF-like lung disease (also known as β-ENaC mouse) was employed in this study, which mimics the sodium hyperabsorption defect in CF and produces a muco-obstructive lung phenotype (Mall et al., 2004). Assessing the protein signatures of lung exosomes from *Scnn1b-tg* mice revealed enrichment of proteins associated with macrophage activation and muco-inflammatory processes. This work highlights the potential for using exosomes as a diagnostic and predictive biomarker for muco-inflammatory lung disease (Mao et al.).

Novel computational models of the human lung integrating image-derived data, pulmonary biomechanics, and structure-function interactions can identify mechanisms and processes involved in lung diseases and injuries, help to develop safe and effective drugs predicting adverse side effects, and support new device design for treatment delivery (Neelakantan et al., 2022). Work presented in an original research article by Liu et al. resulted in construction of a human bronchial tree and development of novel algorithms to model the lungs, which can be employed in simulating and predicting lung dynamics under healthy conditions and various disease states (Liu et al.).

This Research Topic highlights the importance of respiratory models in assessment of therapeutics, understanding of disease pathogenesis, and identification of diagnostic biomarkers. In this Research Topic of articles, animal models of respiratory diseases were primarily used. Novel pharmaceutical strategies were assessed for ALI/ARDS, and the Phe508del CF rat model was found to be responsive to CFTR modulator drugs, providing a basis for their use in future research applications. Lung exosomes were identified to have distinct disease-specific protein signatures, which could be used to diagnose or predict disease outcomes. While animal models remain important in research, the role of computer-based modelling is becoming increasingly relevant, especially as the use of artificial intelligence becomes mainstream in medical applications. Computational models allow us to analyse and process multidimensional datasets from images, texts, and physiological and biomechanical signals to obtain a robust model enhancement. In the future these types of computational models could provide a powerful means for a personalised care of lung disease. Overall, this Research Topic of works highlights the potential to expand the integration of computational modelling alongside traditional biological models, paving the way for further advancements in respiratory pharmacology.
